# 4191 The Role of Suggestibility and Trait Anxiety in Young Adult Alcohol Use

**DOI:** 10.1017/cts.2020.438

**Published:** 2020-07-29

**Authors:** Alexandra Cowand, Melanie Schwandt, Alyssa Schneider, Jodi M. Gilman, Nancy Diazgranados, Vijay A. Ramchandani, Bethany L. Stangl

**Affiliations:** 1 National Institutes of Health; 2 National Institute on Alcohol Abuse and Alcoholism; 3 Yale University; 4 Harvard Medical School

## Abstract

OBJECTIVES/GOALS: The purpose of this study was to investigate how suggestibility and social susceptibility relate to alcohol use in young adult non-dependent alcohol users, and the role of trait anxiety in this relationship. We hypothesized that greater trait anxiety would be associated with higher levels of alcohol misuse, and this would be mediated by suggestibility. METHODS/STUDY POPULATION: Study participants enrolled in the NIAAA screening and assessment protocol completed questionnaires on suggestibility, anxiety, and alcohol use. The Multidimensional Iowa Suggestibility Scale (MISS) is a 95-question self-report assessment of suggestibility. Trait anxiety is assessed with the State Trait Anxiety Inventory-Trait (STAI-T). Alcohol measures included the Alcohol Use Disorder Identification Test (AUDIT). Structured Clinical Interviews for DSM-IV or DSM-5 disorders were conducted, and non-dependent participants (N = 113) were considered. A median split was conducted (median age = 35.1 years), with the focus of this study on the younger individuals (N = 55). RESULTS/ANTICIPATED RESULTS: Initial analyses showed that suggestibility, alcohol misuse, and trait anxiety all had significant positive correlations with one another. To better understand the relationship of peer influence, specifically, with drinking and anxiety, MISS subscale of Peer Conformity was analyzed. MISS total score and Peer Conformity were positively correlated with AUDIT Total as well as STAI-T Score. STAI-T Score was additionally positively correlated with AUDIT Total (all p^2^ = 0.222). We also looked at Peer Conformity in place of MISS Total (R^2^ = 0.213). In both models, only suggestibility measures were significant predictors of harmful alcohol use (p<0.01). DISCUSSION/SIGNIFICANCE OF IMPACT: In young social drinkers, there were significant positive associations between suggestibility, risky alcohol use, and trait anxiety. These results suggest that suggestibility may be a modifiable risk factor for risky alcohol consumption. Future directions include using mediation models to explore the associations between suggestibility, anxiety, and alcohol misuse.

